# Are Motorways Potential Stressors of Roadside Wood Mice (*Apodemus sylvaticus*) Populations?

**DOI:** 10.1371/journal.pone.0091942

**Published:** 2014-03-17

**Authors:** Álvaro Navarro-Castilla, Cristina Mata, Pablo Ruiz-Capillas, Rupert Palme, Juan E. Malo, Isabel Barja

**Affiliations:** 1 Department of Biology, Zoology Unit, University Autónoma of Madrid, Madrid, Spain; 2 Department of Ecology, Terrestrial Ecology Group, University Autónoma of Madrid, Madrid, Spain; 3 Dirección de Innovación y Sostenibilidad, Obrascón Huarte Lain, S.A., Madrid, Spain; 4 Department of Biomedical Sciences/Biochemistry, University of Veterinary Medicine, Vienna, Austria; CNRS, France

## Abstract

Linear infrastructures represent one of the most important human impacts on natural habitats and exert several effects on mammal populations. Motorways are recognized as a major cause of habitat fragmentation and degradation and of biodiversity loss. However, it is unknown whether motorways lead to increased physiological stress reactions in wild animal populations. We analysed faecal corticosterone metabolites (FCM) in wild populations of wood mice (*Apodemus sylvaticus*) living in a well-preserved Mediterranean agro-pastoral woodland at different distances (verge, 500 m and 1000 m) from the AP-51 motorway in Spain. Wood mice were captured with Sherman live traps, and fresh faecal samples from 424 individuals were collected and analyzed in the laboratory. The quantification of FCM was performed by a 5α-pregnane-3β,11β, 21-triol-20-one enzyme immunoassay. Results showed that females had higher FCM levels than males, and these levels were higher in breeding females. In addition, FCM levels were positively correlated with body weight of individuals. Wood mice captured where cattle were present showed higher FCM levels than individuals living where cattle were not detected. FCM levels were higher in non-breeding individuals living close to the motorway compared with FCM levels in those individuals captured further from the motorway. This is the first study showing evidence of the motorways' impact on physiological stress reactions in wild wood mice populations. Understanding how free-living animals are influenced by human interventions could help to understand other subtle changes observed in wild animal populations. Since mice are used world-wide as research models these results could open new perspectives testing human influence on the natural environment and trade-offs of species in degraded ecosystems.

## Introduction

Landscapes are often dissected by an extensive network of linear elements like roads and motorways which occupy huge areas resulting in land use conversion and becoming one of the most extended human impacts in natural habitats [1], [2]. They can affect wild animal populations directly due to road mortality [3], [4] and indirectly, due to the reduction in the connectivity of the landscape because of the habitat fragmentation and to the loss and degradation of habitats [5], [6]. Alterations to light, moisture and wind regimes due to the creation of edges, and pollution from traffic, e.g., light, noise, and chemical, are all perturbations linked to roads accompanying the infrastructure along its whole live [7]. Thus, ecological effects arising from them can determine the viability of local wildlife populations even if their short-term impact is small [3], [5].

Several studies have been performed to determine and understand the effects of roads and motorways on wild animal populations. Most of these studies have focused on medium size to large mammals [3], [8]–[11] and only few studies have been carried out with small mammals [12]–[14]. However, although motorways have negative effects on wild mammalian populations causing a physical and/or behavioral barrier effect [15], no studies have documented their effects on other aspects, e.g. the effect of motorways on physiological stress reactions in wild mammal populations is unknown.

For the assessment of physiological effects, glucocorticoids (GC) have been used as indicators of stress [16]–[21]. Especially in wildlife studies, faecal cortisol/corticosterone metabolites (FCM) have been utilized as a suitable, non-invasive measure of GC levels to evaluate responses during stressful stimuli and the endocrine status in an increasing number of mammalian species [21]–[24].While short-term GC secretion is related to animal adaptive responses to a stressor [25], chronically elevated and prolonged high GC levels caused by environmental or human perturbations can lead to serious consequences on reproduction, the immune function, growth, survival and fitness [17], [26]–[29].Different studies performed in natural conditions have correlated GC levels to human perturbations [23], [30]–[35]. So, since motorways are human made interventions and involve the modification of natural ecosystems, they could be perceived by animals as stressors evoking a stress response in wild populations. Therefore, the aim of the present study was to test the hypothesis that human disturbance by motorways is a potential stressor that may affect GC concentrations causing physiological stress reactions in small mammals like the wood mouse (*Apodemus sylvaticus*). Most ecological effects are continuous along a road but their extension or impact over wild populations depend on the closeness to the road, and this significant impact varies from a few meters to a few kilometers [36], [37]. Therefore, if wood mice perceive vehicles as a real disturbance, then we predict that the FCM levels would vary in relation to the distance to the motorway. We would expect higher FCM levels in those individuals living closer to the motorway subjected to a major degree of environmental change and human disturbance due to traffic. However, if traffic is not a direct disturbance and roads are favorable habitats, FCM levels should be similar regardless the distance to the motorway.

## Materials and Methods

### Ethics Statement

Manipulations of animals were done in compliance with the European Communities Council Directive 86/609/EEC for animal experiments and were carried out under the permit of Dirección General del Medio Ambiente de la Junta de Castilla y León (Reference: CML/mjg; File: EP/CYL/424/2009). The responsible of the Institutional Animal Care and Use Committee from the Universidad Autónoma de Madrid was consulted about the need of a specific permit to develop the experiments. Such permission was considered unnecessary, given that captures did not involve endangered nor protected species and capture and release did not include invasive procedures or others that would negatively affect animal welfare. Capture procedures have been detailed in the animal trapping and data collection in the field section.

### Study Area

The study took place along a stretch of the AP-51 motorway in Ávila (central Spain) and it was carried out under the permit issued by the Dirección General del Medio Ambiente, Junta de Castilla y León (Reference: CML/mjg EP/CYL/424/2009) with permission from the land proprietors of El Tabladillo estate and landowners from Mediana de Voltoya and Ojos Albos. The road is fenced and it has two lanes in each direction, with a mean traffic volume of 8396 vehicles per day. The study area is included in the European Natura 2000 Network as a Site of Community Importance (SCI ES-4110103 Encinares de los ríos, Adaja and Voltoya). The surrounding environment is dominated by holm oak (*Quercus ilex*) woodlands, mixed with open holm oak grazing woodlands devoted to low intensity livestock ranching, fallow areas and scrub and unirrigated cereal crops. The study area has a typical Mediterranean climate with an annual mean temperature of 10°C, a mean annual precipitation of 364 mm and two-month long dry season in July–August. The location ranges from 1050 to 1250 m above sea level.

### Animal Trapping and Data Collection in the Field

Live trapping was conducted in February and April of 2010 along five kilometres of the AP-51 (UTM 30 T 373259 4510571– 30 T 368053 4507625) where four 1250 m sectors were selected. In each sector, three bands were established at increasing distances (0–50 m, 500 m and 1000 m) in line with the road [38]. The three distance bands were sampled simultaneously to avoid the effects of weather conditions and moonlight on small mammals' activity [39], [40]. Thirty Sherman live traps (23×8×9 cm) were set up in each band with 10–15 m of separation between them, for three consecutive nights and with a total effort of 2160 traps-night. Traps were reviewed twice-daily every 8–10 hours, at dawn and dusk, to minimize the time that animals were kept inside. Bread fried in rancid oil was used as bait and cotton nesting material was provided inside each trap as bedding to protect captives from low temperatures. In addition, traps were hidden under vegetation cover to protect animals from adverse weather conditions and to avoid detection by predators. Captured animals were marked with subcutaneous sterile Trovan ID100 passive transponders to identify recaptures in order to achieve sample independence. Individuals were weighted with a hand-held scale. Sex and breeding condition of individuals were determined following the method of [41]. The distance between the clitoris and anus in females is smaller than the distance between the penis and anus in males. Breeding adult males present the testicles enlarged quite markedly and usually descend into the scrotal sac while in breeding adult females the nipples on the abdomen and thorax are noticeable and the vaginal membrane appears perforated. To reduce disturbance to animals, all captured individuals were handled as fast as possible and they were released in the same place of capture. Traps were cleaned with clean water to avoid the mixing of different samples and then reactivated.

Since vegetation characteristics and cattle activity may affect small mammals' behaviour and/or abundance [42]–[44], these variables were recorded as well as the distance-band. Vegetation cover and height were measured around each trap (area of 5 m radius) by two experienced observers. The plant cover (in %) was visually estimated, and the mean height of the herbaceous and shrub stratum (in cm) was measured by placing a stick vertically on the ground. Cattle activity (presence or grazed field) was coded as one when there were signs of cattle activity and otherwise it was recorded as zero.

### Faecal Samples Collection

Faeces were collected from traps where a single wood mouse was captured. We only collected fresh faecal samples (i.e. with a soft texture and not dried) in order to prevent degradation of steroids by microorganisms [45]. To avoid cross contamination faecal samples from traps where urine was detected were excluded. FCM appear in faeces about 10 h after an ACTH injection (median: 10 h, range: 8–12 h; [46], so traps were reviewed within 10 hours to avoid any possible effect of the capture itself on FCM levels. Circadian rhythm is supposed to have an important influence in excretion patterns [46], [47], so we considered only the fresh samples collected during the early morning checking. Faecal samples were stored in the freezer at −20°C until analysis.

### Measurement of Faecal Corticosterone Metabolites (FCM)

Extraction of FCM from the faeces was performed as described previously [47], [48]. Briefly, each faecal sample was homogenized and 0.05 g were weighed and mixed with 1 ml of 80% methanol in an eppendorf tube. Samples were shaken for 30 min on a multivortex and then centrifuged (15 min at 2500 *g*). Supernatants obtained were diluted 1∶10 with assay buffer and stored at −20°C until analysis. FCM were analyzed using an already established 5α-pregnane-3β,11β,21-triol-20-one enzyme immunoassay (EIA) measuring metabolites with a 5α-3β,11β diol structure. This method was previously developed and validated for mice by [46], [47].

### Statistical Analyses

We used a General Linear Model (GLM) to analyze factors influencing FCM levels. The response variable, i.e. concentrations of FCM, was log_10_ transformed to reach normal distribution and homocedasticity. Categorical predictors were band's distance (0–50 m/500 m/1000 m), sex (male/female), breeding condition (breeding/non-breeding), and cattle (presence or habitat grazed/no signs), while weight of individuals was fitted as covariable. Plant cover and height were not included in the statistical model since their values were mainly related to cattle grazing as denoted by exploratory analyses. Thus, ungrazed sites showed a denser and taller grass and shrub layer than grazed ones. Differences in FCM levels between groups were analyzed for significant interactions in the GLM through Tukey's honestly significant difference (HSD) post-hoc tests carried out on their marginal means. Results were considered significant at p<0.05. Data are given as mean ± standard error (SE). We used the SPSS 15.0 statistical software (SPSS Inc, Chicago, IL, U.S.A.) to perform the GLM, and Tukey HSD tests were computed in a worksheet according to formulae [49].

## Results

FCM levels were analyzed in fresh faecal samples from a total of 424 different wood mice (Table 1). The statistical model (Table 2) showed that significant factors explaining the variation found in FCM concentrations were sex, breeding condition, body weight of individuals and cattle. In addition, the interactions between sex*breeding condition and band*breeding condition resulted in a significant influence on FCM levels. Other interactions were not statistically significant.

**Table 1:** Number of captures in the different distance bands in relation to sex and breeding condition of individuals.

	Distance bands					
	0–50 m		500 m		1000 m	
Number of captures	187		94		143	
Males/Females	88/99		42/52		82/61	
Breeding/Non-breeding	10/78	29/70	4/38	11/41	2/80	12/49

**Table 2:** Results of the General Linear Model testing the effects of individual and environmental variables on faecal corticosterone metabolites in wood mouse.

Factor	*F*	df	*P*
Band	0.326	2	0.722
Sex	11.730	1	0.001
Breeding condition	11.711	1	0.001
Weight of individuals	14.945	1	0.000
Cattle	3.944	1	0.048
Sex * Breeding condition	4.786	1	0.029
Band*Breeding condition	5.166	2	0.006
Band*Sex	2.241	2	0.108
Band*Cattle	1.608	1	0.205
Cattle*Sex	2.004	1	0.158
Cattle*Breeding condition	2.174	1	0.141
Band*Cattle*Sex	0.323	1	0.570
Band*Cattle*Breeding condition	0.035	1	0.852
Band*Sex*Breeding condition	0.634	2	0.531
Cattle*Sex*Breeding condition	3.355	1	0.068

**Figure 1:**
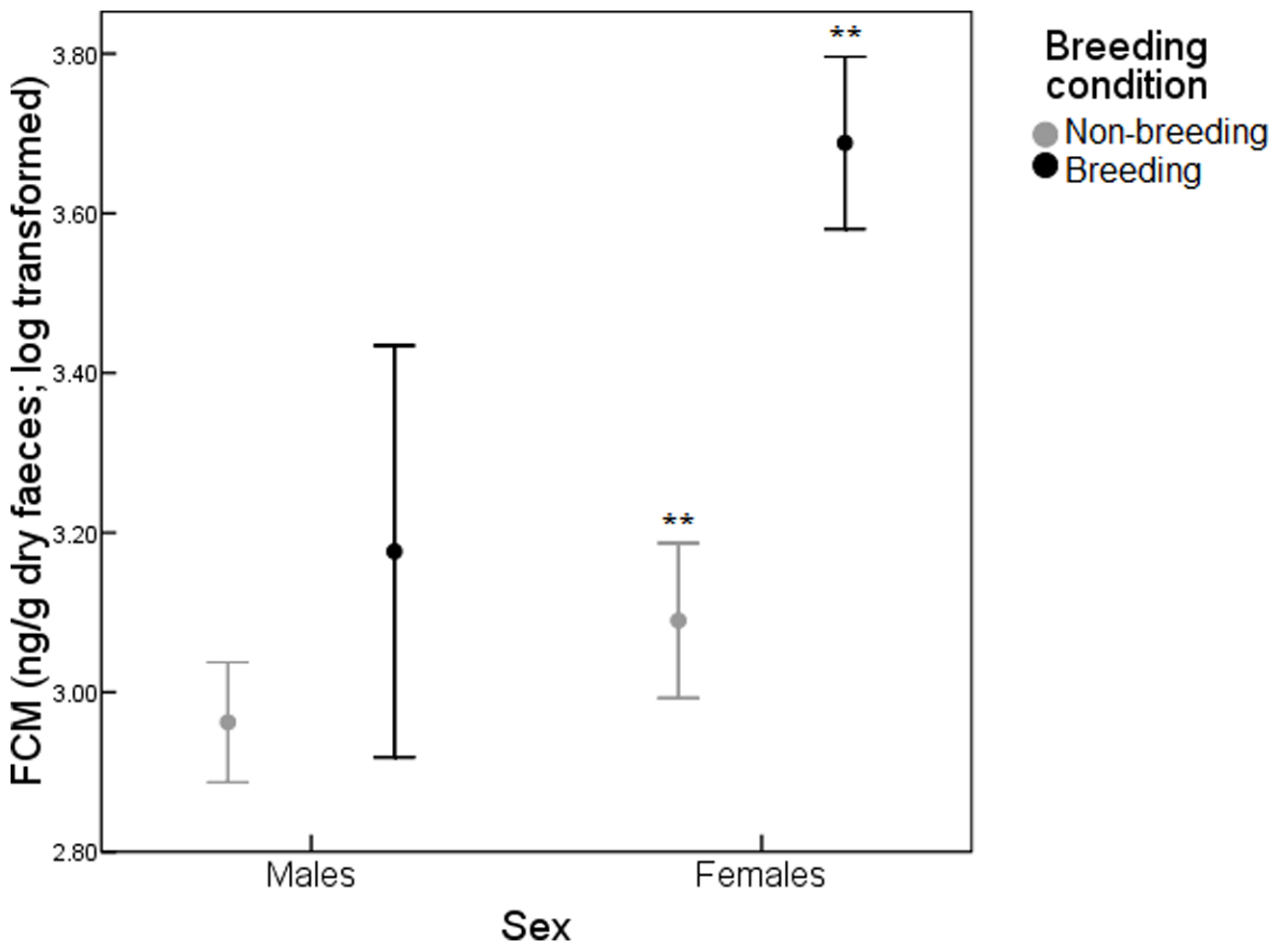
Comparison of FCM concentrations (ng/g dry faeces; mean ± SE) according to breeding condition for males and females. Asterisks indicate significant differences (**p<0.01).

FCM levels differed between sexes, concentrations of FCM were higher in females (4467±485 ng/g dry faeces) than in males (2057±235 ng/g dry faeces) (*F*_1,424_ = 11.73, *P* = 0.001). Breeding individuals showed higher FCM levels (6609±978 ng/g dry faeces) compared to the non-breeding ones (2601±255 ng/g dry faeces) (*F*_1,424_ = 11.71, *P* = 0.001). Nevertheless, the interaction between sex*breeding condition (*F*_1,424_ = 4.79, *P* = 0.029) showed that differences due to breeding condition were only significant for females (Tukey's HSD, *P*<0.01), but not for males (Tukey's HSD, *P*>0.05) (Fig. 1). In addition, FCM correlated positively with body weight of animals (*F*_1,424_ = 14.95, *P*<0.0001). Cattle had a significant effect on FCM levels (*F*_1,424_ = 3.94, *P* = 0.048), individuals captured where cattle (presence or habitat grazed) were detected shown higher FCM levels (3779±451 ng/g dry faeces) than individuals captured where no signs of cattle were recorded (3042±344 ng/g dry faeces).

**Figure 2:**
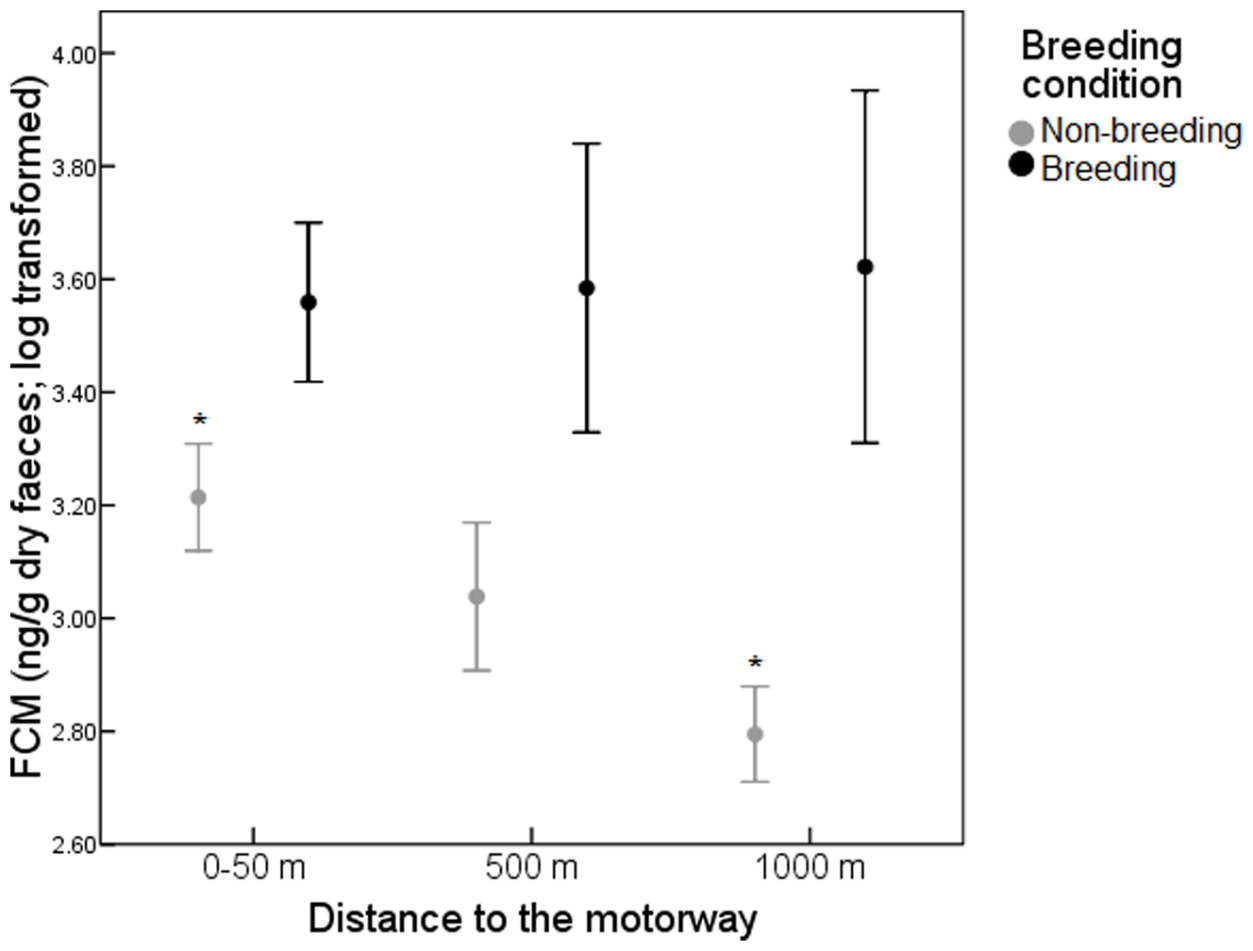
Log-transformed concentrations (mean ± SE) of faecal corticosterone metabolites (FCM, ng/g dry faeces) in breeding and non-breeding individuals in relation to the distance to the motorway. Significant differences are indicated by asterisks (*p<0.05).

The significant interaction between band*breeding condition showed that FCM levels varied according to the distance to the motorway, however, significant differences between distance-bands depended on breeding condition (Fig. 2). FCM levels were similar in the three distance-bands for breeding individuals (Tukey's HSD, *P*>0.05) but they showed significantly higher values in the non breeding individuals from the 0–50 m band compared with those captured in the 1000 m band (Tukey's HSD, *P*<0.05). FCM values for non-breeding individuals captured at 500 m were midway between those of individuals captured beside the road and at the 1000 m band.

## Discussion

Glucocorticoids relationship with sex and breeding condition has yielded ambiguous results [46], [47], [50]–[52]. We found differences in FCM levels between both sexes, females showing higher levels of FCM than males. The same situation was reported for other rodent species [46], [53] and this difference could be partly due to differences in the metabolism of glucocorticoids between both sexes [47]. Higher glucocorticoid levels during breeding season have been reported in several studies performed in different mammalian species [23], [54]–[57]. However, in our case we only found this pattern for females but not for males, probably because the low sample size of males. Nevertheless, a possible explanation could be that testosterone in breeding males can suppress glucocorticoids, whereas high oestrogen levels during lactation and pregnancy should increase them [58]. Glucocorticoids play an important role in metabolism [59] and it is known that breeding females undergo a series of metabolic changes during pregnancy and lactation due to their maternal investment [60]–[62]. In addition, reproduction costs reduce longevity in mice and other species [63], [64], especially in females [65]. So, reproduction could be probably not particularly stressful for males since their investment is usually limited to fertilization, whereas socio-sexual aggression may be a normal component of female's cost of reproduction and females, through gestation and lactation, invest heavily in the offspring's survival. Therefore, the difference found in FCM levels between males and females and during breeding could be explained by the mix of different metabolism patterns and their different constraints on reproduction.

We found that FCM levels were positively related with body weight of individuals. Since in this species weight of individuals is closely related with animal's age [41], our results suggest adult individuals showing higher levels of physiological stress reactions than young individuals. Elevated glucocorticoid secretion promotes physiological and behavioral responses that enhance an adult animal's ability to cope with stressors and other challenges to homeostasis [66], whereas less well understood is whether similar glucocorticoid-mediated responses occur during other different stages of life [67]. These results could be explained by age-related alterations [68]. Alternatively this difference found in FCM levels between young and adult animals could also be due to differences in metabolism and excretion of glucocorticoids rather than differences in plasma corticosterone levels [22].

The effect of cattle on FCM levels could be explained because grazing by herbivores reduces vegetative cover drastically [69], [70] and this has an important impact on small mammal communities [71]–[74]. Small mammals perceive dense vegetation as good protection [75] since the reduction of vegetation cover exposes small mammals to increased predation risk [44], [76], [77]. Thus, our results suggest that habitats without cattle, therefore with more grass and shrub cover, could be considered as better quality habitats with a higher protection from predators and therefore lower FCM values.

The higher FCM values found in breeding individuals in the three distance-bands showed that breeding condition has a huge influence on FCM as we have discussed above. These results suggest that the effect of motorway on FCM could be less important when there are other stronger factors like breeding status affecting to the individuals. However, the higher FCM levels found in non-breeding individuals living close to the motorway could be explained by the alteration of the natural habitat and the perturbation of the motorway. An effect that could be diluted as distance to the motorway increases, with levels still higher at 500 m than at 1000 m. Road maintenance and traffic aggravate edge effects on the surrounding environment by noise and pollution. Vehicle's emissions cause atmospheric pollution and consequent ecological effects for wildlife [78] and traffic noise is known to be an important source of disturbance [79], [80]. However, visual disturbance, the barrier effect and pollutants extended outward only a short distance compared with traffic noise that spreads far into the environment. Several studies have shown that effect-distances are sensitive to traffic density, traffic speed or the type of habitat being higher the effects in the proximity of roads and varying from 300 m to 900 m [81], [82]. In our case, both traffic (density and speed) and habitat type was similar along the five kilometres selected, so this distance effect of traffic noise could explain the variation found in FCM levels at different distances from the motorway. Many possible reasons exist for the effects of traffic noise on wildlife, likely hypotheses include hearing loss, altered behavior, interference with communication during breeding activities, differential sensitivity to different frequencies, and deleterious effects on food supply or other habitat attributes [81]–[84]. Taken together, our results suggest that the negative effects of motorways, through the barrier effect or the traffic noise, seem to be an important factor of perturbation for wildlife communities evoking an increase in FCM levels.

According to our results, there is a first evidence of motorways increasing stress reactions in a small mammal species. Wood mouse is known by its great ability to use a wide range of open habitats including urban habitats, forest, meadows, forest, grasslands and agricultural crops [85]–[89] and is, therefore, less likely to be dramatically affected by environmental changes. However, several studies have shown that small mammals are hardly affected by the barrier effect of roads [13], [14] and our results demonstrate that in this species motorways are acting like a stressor factor, and even this could be for other animal species too. Our study has implications for the effectiveness of different possible mitigation strategies to reduce the impact of roads on mammal species being the major conservation objective focused in the conservation of large roadless areas [90]. To our knowledge, this is the first attempt to test the physiological stress reaction in wood mice caused by the perturbation of motorways. Our results are of great concern due to the high proliferation of roads in the landscape over recent decades. In combination with other studies demonstrating complex and negative effects of roads on other species, affecting populations' viability, our results might be relevant since FCM levels may indicate that an animal is stressed even if obvious behavioral changes cannot be detected [91]. These results shown that FCM measurement can provide a powerful tool for addressing and evaluating the impacts of human disturbance on wildlife populations. However, the biological responses evoked to cope with a stressor vary among species, age, experience and physiological status [92], [93]. So, further research should be performed in order to study this human-based stressor in other wild animal species.
